# Community-Engaged Approaches to Cervical Cancer Prevention and Control in Sub-Saharan Africa: A Scoping Review

**DOI:** 10.3389/fgwh.2021.697607

**Published:** 2021-07-19

**Authors:** Magdiel A. Habila, Linda Jepkoech Kimaru, Namoonga Mantina, Dora Yesenia Valencia, D. Jean McClelland, Jonah Musa, Purnima Madhivanan, Atiene Sagay, Elizabeth T. Jacobs

**Affiliations:** ^1^Department of Epidemiology and Biostatistics, University of Arizona, Tucson, AZ, United States; ^2^Department of Health and Pharmaceutical Outcomes, University of Arizona, Tucson, AZ, United States; ^3^Department of Health Promotion Sciences, University of Arizona, Tucson, AZ, United States; ^4^Clinical Translational Sciences, College of Medicine, University of Arizona Health Sciences, Tucson, AZ, United States; ^5^Health Sciences Library, University of Arizona, Tucson, AZ, United States; ^6^Department of Obstetrics and Gynecology, Jos University Teaching Hospital, Jos, Nigeria; ^7^Department of Preventive Medicine, Division of Cancer Epidemiology and Prevention, Northwestern University, Chicago, IL, United States

**Keywords:** cervical cancer, Sub-Saharan Africa, community engagement, cervical cancer prevention and control, review

## Abstract

**Background:** Cervical cancer remains one of the top causes of cancer mortality among African women. Cervical cancer screening and early detection and treatment of precancer is one of the evidence-based interventions to reduce incidence and mortality. The application of community-based participatory research (CBPR) has been used in the United States to improve participation in screening and reduce cervical cancer disparities. However, it is unclear whether these engaged approaches have been used in sub-Saharan African to address disparities related to cervical cancer mortality.

**Objectives:** Highlight community engagement in cervical cancer prevention and control in Sub-Saharan Africa (SSA), describe the community engagement efforts that are currently being used, and to describe the best practices for community engagement toward the end-goal of cervical cancer prevention and control.

**Methods:** We searched PubMed, Embase, CINHAL, African Journals Online (AJOL), and African Index Medicus-WHO from inception until June 8, 2020. After screening 620 titles and abstracts, and reviewing 56 full-text articles according to inclusion and exclusion criteria, 9 articles met the selection criteria and were included. Relevant data variables were extracted from the included articles and a narrative synthesis was performed.

**Results:** Between 2005 and 2019, 9 articles describing research in Ghana, Kenya, Zambia, Senegal, South Africa, and Nigeria were included. These articles described work that largely took place in rural settings predominantly among women age 15–65 years. Leveraging community networks such as community health workers, religious organizations, traditional leaders, and educational institutions increased awareness of cervical cancer. Working within existing social structures and training community members through the research effort were promising methods for addressing the disparities in cervical cancer incidence and mortality among communities.

**Discussion:** The findings of this scoping review have contributed to the understanding of which novel approaches to community-based practices can be used to address cervical cancer disparities among SSA communities that carry a disproportionate disease burden. Community engagement in the research process, while effortful, has shown to be beneficial to researchers and to the communities that they serve, and provides valuable next steps in the effort to address cervical cancer disparities in SSA.

## Introduction

Cervical cancer is the leading cause of death in women globally (1). This disease is caused by persistent infection with oncogenic strains of the human papillomavirus (HPV). Aside from avoiding sexual activity, the HPV vaccination of young girls before sexual activity is an effective intervention for primary prevention of cervical cancer (2). The uptake of the HPV vaccine and organized cervical cancer screening have decreased cervical cancer incidence in developed countries (3). In the United States, there has been a significant decrease in cervical cancer incidence since the introduction of HPV vaccination and screening (4, 5). However, cervical cancer is the leading cause of cancer-related deaths in African women, where the estimated incidence rate was 139.6 per 100,000 women and the mortality was an estimated 94.1 per 100,000 in 2018 (6).

The high incidence of cervical cancer in Sub-Saharan Africa is a complex issue with social, cultural, and economic components (2). The HPV vaccine is costly to obtain and has many strict requirements for preservation that make it a challenge to store and distribute in many low- and middle-income countries (LMICs) (2, 7). Perceptions that the vaccine will encourage socially unacceptable behavior in children and youth who receive the HPV vaccine and other forms of misinformation serve as local social barriers to increased uptake of this vaccine (8–10). Moreover, another major barrier is the lack of knowledge about HPV vaccination and cervical cancer prevention among the general population in LMICs (7, 9). The combination of all these factors detract from the efforts to effectively prevent cervical cancer in low-resource and high-risk settings.

Given the complexity of the barriers surrounding cervical cancer prevention and control, a new perspective on ways to improve knowledge about the risk associated with cervical cancer and effective, scientifically sound prevention methods are essential in low-resource communities. The premise of community based participatory research (CBPR) is that community members are active participants in the research process from the development of the research question to dissemination of study findings, which results in more education and empowerment within the community (11). CBPR is an approach that has been used to address the needs of underserved communities in the United States for many years (12, 13). It is unclear whether CBPR has been used in Sub-Saharan Africa, and if it has been effective in addressing the lack of knowledge about cervical cancer in African communities. This approach is particularly useful within underserved communities because it encourages the community to be partners in the research endeavor, rather than research subjects—which minimizes the effect of existing distrust of researchers that often exists within the communities (11, 13–15).

CBPR has proved a useful tool in increasing knowledge about the prevention and control in underserved communities living in the United States that experience disproportionate breast and prostate cancer burdens (13, 15). However, there are gaps in our understanding of the application of CBPR as framework for improving cervical cancer prevention and control efforts in Sub-Saharan Africa. The findings of this review provide a summary of the use of CBPR and similar community engaged approaches in cervical cancer prevention and control in Sub-Saharan African communities, provide insight on the way “community” has been defined in previous studies, and generate hypotheses for new methods of studying and understanding cervical cancer prevention and control in these communities.

A scoping review is an ideal tool for describing what types of community-engaged approaches have been used in Sub-Saharan Africa to address the high incidence of cervical cancer, and to determine which strategies were most effective in increasing knowledge about cervical cancer in this population. Understanding how CBPR practices can be leveraged to decrease the incidence of cervical cancer and its associated costs while also engaging and empowering community members toward healthier lives is an important step in decreasing the disproportional disease burden that women in LMICs experience. The objectives of this review are 3-fold: describe community engagement efforts in cervical cancer prevention and control in SSA, to identify the areas of cervical cancer research where community engagement has been used, and to describe the best practices for community engagement toward the end-goal of cervical cancer prevention and control.

## Methods

Community engagement, as was defined above, usually signifies including community members in research, but within a limited scope. For example, community members are often invited to share their experiences in focus groups and/or interviews and are asked to help with the recruitment of other potential participants. As it stands, community engagement is a common tool used by research groups working with disease prevention and control. Consequently, due to its limited scope, community engagement is often not enough to foster cyclical and iterative research processes between researchers and the community, and to achieve intervention sustainability. For this reason, CBPR principles can act as a tool to enhance community engagement by guiding this practice to the next level, toward true community-based participatory research (11).

CBPR is a specific type of community-engaged research. For this review, a broad definition of community engagement was adapted to capture all the types of community engagement that have been used in research in Sub-Saharan Africa, not just studies that defined themselves as CBPR studies. Community engagement was defined as “research efforts that include community members in the development of a research question, interpretation of study results, and/or implementation of research interventions or findings” (16).

The authors conducted this review in accordance with a protocol that can be accessed here https://www.researchsquare.com/article/rs-37012/v1. This review is not registered in PROSPERO because scoping reviews are no longer eligible for registration.

### Community-Based Participatory Research Framework

The purpose of this review is to describe community-engaged approaches to cervical cancer prevention and control methods. Therefore, the Community-Based Participatory Research (CBPR) framework was used to examine the strengths of previous studies that have used this approach, and to identify themes effective strategies that future studies can employ to address the disparities in cervical cancer outcomes in SSA. The CBPR framework consists of eight principles: each describing an aspect of the relationship between the researcher(s) and the research participants. The principles are: recognizing community as a unit of identity, builds on strengths and resources within the community, facilitates collaborative partnerships in all phases of the research, integrates knowledge and action for mutual benefit of all partners, promotes a co-learning and empowering process that attends to social inequities, involves a cyclic and iterative process, addressed health from both positive and ecological perspectives, and disseminates findings and knowledge gained to all partners (11). These principles emphasize the importance of partnership between the researchers and the community, leading to attention to and action addressing community needs, and implementation and dissemination of research findings within community networks. The integration of these aspects of the research effort will support sustainable and equitable change within communities. This review uses this understanding to examine previous literature on cervical cancer prevention and control in SSA communities.

### Eligibility Criteria

Inclusion criteria for this review included articles that pertained to women who were at risk for cervical cancer or were diagnosed with cervical cancer, articles that related to cervical cancer prevention and/or control, studies that took place in sub-Saharan Africa, and included a reference to community engagement during research activities. Inclusion criteria were not restricted by study design because community-engaged research can be conducted in a variety of research settings and in a variety of ways. Studies were excluded if they were not published in English, were commentaries, conference abstracts, or editorial reviews. Studies where community members were participants in the study but were not included in any other aspect of the study were excluded because this was not considered “engagement” according to the definition stated above. All studies that did not take place in a sub-Saharan African country were excluded to obtain a collection of studies that were specific to sub-Saharan Africa. Finally, studies in which the researchers were focused on HPV testing as the primary outcome of the study were excluded because we sought to describe efforts specific to cervical cancer prevention, rather than on the prevention of its precursors.

### Information Sources

#### Primary and Secondary Searches

The primary searches for this review were conducted in PubMed, Embase, and CINHAL. No time restrictions were placed on these databases, and papers that were published in these databases at the final search date of June 8, 2020, were included. The search string for this review was developed in collaboration with an experienced librarian and is available in the Supplementary Material. Our search terms consisted of three concepts including, community-based research, sub-Saharan Africa, and cervical cancer.

Secondary searches were also conducted in African Journals Online (AJOL) and African Index Medicus-WHO, regional databases that may not have been covered by larger Western databases. The National Technical Information Service (NTIS) database, OpenGrey, and Web of Science were searched as sources of gray literature not captured by the larger databases. Date and language restrictions were not placed on these databases. All included studies were available for review in English. To ensure consistency, the same search string was used for both primary and secondary searches.

#### Review of Articles

Once the primary and secondary searches were completed, all included articles were managed with Covidence (Covidence systematic review software, Veritas Health Innovation, Melbourne, Australia. Available at www.covidence.org) to be de-duplicated. Deduplication was completed based on title, authors, and year of publication. Then, the title and abstract of each article was screened by two independent review authors, and discrepancies were resolved by a third reviewer. All included articles then underwent full-text review conducted by two independent study reviewers, with a third reviewer settling discrepancies.

##### Data Charting Process

Once the full-text review was completed, data extraction began using the standardized form within Covidence. Each study was reviewed by two review authors, and any discrepancies were reconciled by a third review author. The data extraction form was developed based on previous literature and the research questions. The study authors agreed on the relevant variables for abstraction from each of the selected articles included in this review.

##### Data Items

The data items collected from the study were identified and included based on prior knowledge established in the literature on cervical cancer and community-engaged research and based on the research questions that the review team sought to answer. From each study, data were collected on the country of the study setting, urban or rural designation as stated in the study, type of study (qualitative or quantitative), study design, theoretical framework utilized in the study, description of the type of community engagement that was used, study inclusion and exclusion criteria, sex and age of participants, location and method of participant recruitment, location of community engagement, language(s) of community engagement, types of data collected in the study, a description of the intervention used, the community members involved in study design/implementation, and study outcomes.

##### Synthesis of Results and Critical Appraisal Within Sources of Evidence

A spreadsheet was created and used to managed the data variables extracted from the included studies. Concordant and discordant conclusions were determined and resolved in Covidence. All data were summarized in tables and described in narrative form. Critical appraisal of the sources of evidence was not conducted because this is not typically conducted in scoping reviews (17).

## Results

### Study Characteristics

From the inception of the databases to the final search on June 8, 2020, the searches yielded 620 studies, which after removal of duplicates included 556 individual articles for title and abstract screening. Title and abstract screening excluded 500 studies that did not mention community engagement, taking place in sub-Saharan Africa, or cervical cancer. Fifty-six studies were reviewed at the full-text stage. Of these, 20 articles (36%) were excluded because they did not describe a method for community engagement according to the CBPR principles described above. Additionally, 7 articles (13%) did not focus on cervical cancer prevention and control, 4 articles (7%) were not conducted in sub-Saharan Africa, 4 articles (7%) were commentaries. Finally, 12 articles (21%) were excluded because they were conference abstracts or did not report results of a research study. Nine articles (16%) selected for inclusion constituted the final analysis sample for this narrative review.

**Figure 1:**
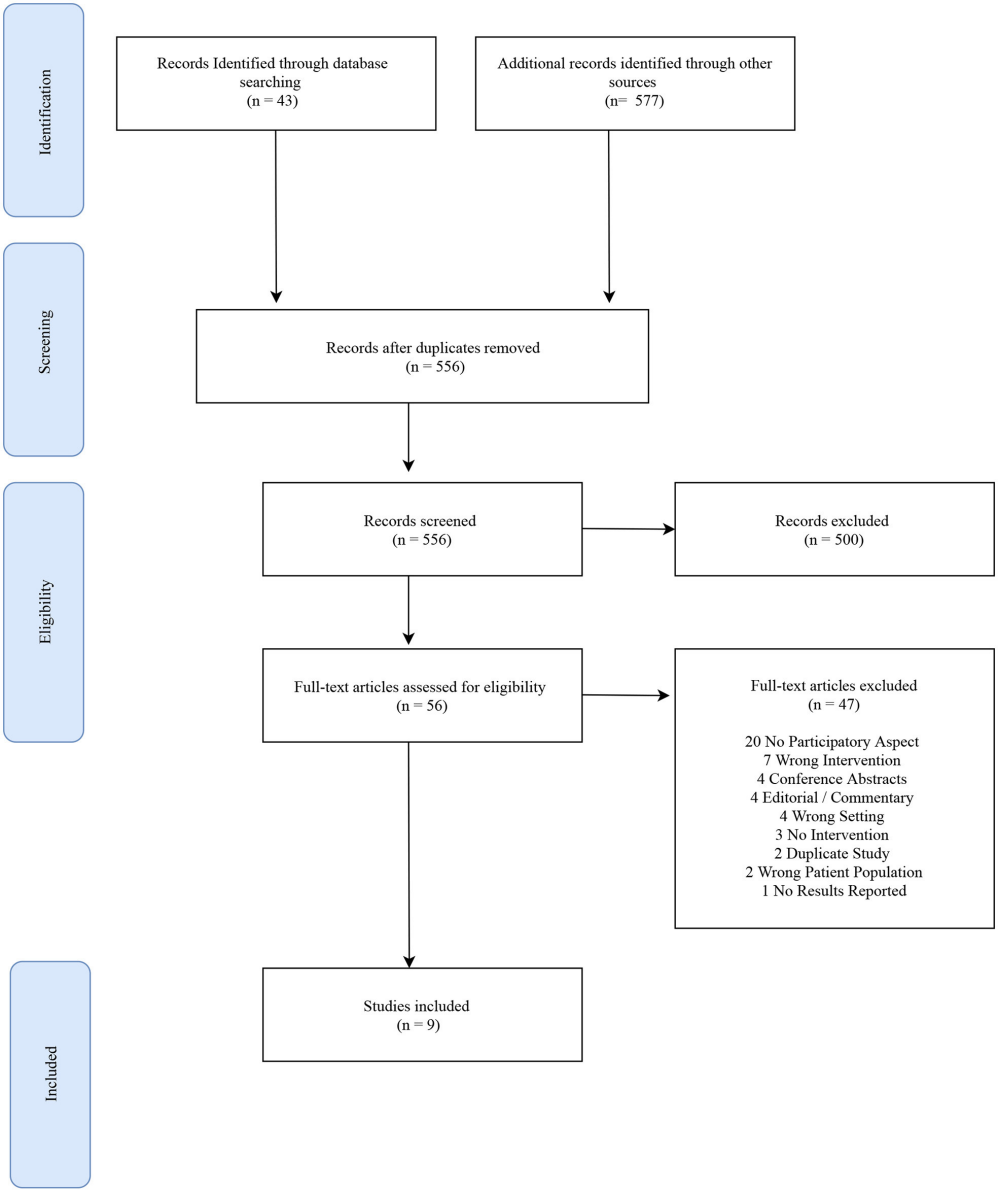
Flow diagram of the source selection process.

Figure 1 shows the results of the screening and inclusion process according to the previously stated inclusion and exclusion criteria. Nine studies were included in this review which were published between 2005 and 2019. Studies were conducted in 6 countries: Ghana (*n* = 1), Kenya (*n* = 3), Nigeria (*n* = 2), Senegal (*n* = 1), South Africa (*n* = 1), and Zambia (*n* = 1). Sixty-seven percent of the studies took place in rural settings. The age range of participants across all studies was 15–65 years old. Additional study characteristics and outcomes are displayed in Table 1.

**Table 1:** Description of study characteristics of included articles.

**References**	**Population**	**Country**	**Study setting**	**Study design**	**Purpose of the study**	**Comment on use of community engagement strategies**	**Results**
Abril et al. (18)	76 women	Senegal	Rural	Qualitative	Evaluate the communication infrastructure, knowledge, and attitudes of cervical cancer in Senegalese women.	Not described.	Overall, women had minimal knowledge of cervical cancer. Women preferred that health information be communicated in face-to-face interactions. Friends and male physicians were seen to be reliable sources of information.
Aja et al. (19)	30 women aged 26–51+ years	Nigeria	Urban	Cohort study	Describe ways to engage church-based women's groups as a vehicle for developing and delivering culturally relevant women's health information, including cervical cancer prevention.	Churches were viewed as a resource within the local communities because they can serve as a venue for delivery of health information.	30 faith leaders from 15 churches participated in the 2-day health workshop. Thirteen churches submitted requests to their leadership to implement the workshop in their local congregations. At 3 months follow-up, three churches had hosted a workshop for their local congregation.
Awua et al. (20)	410 women aged 15–65 years	Ghana	Rural	Cross-sectional study	Describe the development of a community-based cervical cancer screening program in response to low uptake of the current clinic-based screening strategy.	Screening was conducted in the community compared to screening based in the clinical setting.	Researchers had a 96.1% response rate to the community-based screening strategy. Some women preferred self-specimen collection method because of pain from and/or fear of sample collection by a health professional. Others preferred health personnel specimen collection because health professionals were more knowledgeable and experienced.
Chigbu et al. (21)	2,313 women aged 31–60 years	Nigeria	Rural	Cross-sectional study	Assess the motivations and preferences of cervical cancer screening by visual inspection with acetic acid (VIA) among Nigerian women.	Community engagement strategies were utilized to improve utilization of cervical cancer screening services among women.	Participants identified that the common motivational factors for cervical cancer screenings were support from their husbands, and support from community leaders. Most women expected immediate results from screening tests and immediate treatment for any irregularities.
Huchko et al. (22)	4,944 Women aged 25–65 years	Kenya	Rural	Cluster randomized trial	Compare utilization of HPV-based cervical cancer screening when they were offered in community health campaigns to screening in government clinics	Community health campaigns are a high impact and low-cost method of delivering community-based healthcare.	Community health campaigns reached more women for cervical cancer screenings than clinic-based campaigns. This was particularly true for those in hard-to-reach rural areas.
Kapambwe et al. (23)	8,399 women	Zambia	Rural	Cross-sectional study	To assess the role of traditional chiefs in promoting village-based cervical cancer screenings.	Zambia has 244 Chiefdoms that are officially recognized in the country's Constitution. Chiefs have a heavy influence on their constituents and on the activities that take place in their territories.	83.9% of women who attended community events promoted by local chiefs were screened for cancer. Of those who tested positive, 65.8% received same-day treatment.
Mosavel et al. (24)	181 youth, mothers, educators, support staff, community stakeholders	South Africa	Urban	Cross-sectional study	Evaluate the feasibility of an adolescent focused cervical cancer prevention program.	The political history of South Africa has cultivated an environment of community engagement, and therefore this project is a natural fit for the use of CBPR.	There was an ethical concern of focusing only on young girls when older women have a higher risk of cervical cancer in South Africa. Health interventions need to account for cultural, historical, and economic conditions of the country, all of which inform health-seeking behaviors.
Podolak et al. (25)	107 women, men, community health volunteers, local leaders, and professionals	Kenya	Rural & Urban	Qualitative	Understand the decision-making process of local leaders in implementing a cervical cancer screening program.	Researchers utilized participatory action research methodology to engage local experts.	Self-sampling was socially acceptable among women. Local leaders identified that paying for cancer screenings is a barrier to the majority of women.
Swanson et al. (26)	255 women aged 25–65 years	Kenya	Rural	Cross-sectional study	Evaluate the uptake and acceptability of community health campaigns as a method of providing cancer screening and to report cancer prevalence and completion of treatment.	Utilizing community health campaigns helps overcome barriers associated with clinic-based screening.	There was a positive response to self-collection cervical cancer screening among community members. Most women preferred to receive their results by text. Half of the women who tested positive for HPV received treatment within a month.

#### Primary Objectives

*Objective 1*: *Describe community engagement efforts in cervical cancer prevention and control in SSA*.

Two articles including Mosavel et al. (24) and Podolak et al. (25) out of the nine included in the review intentionally used the CBPR framework as the foundation for their cervical cancer prevention and control efforts (24–26).

These articles indicated that researchers have leveraged community networks to achieve increased knowledge about and prevention of cervical cancer by working with community health workers, religious organizations, tribal leaders, and school-aged students and their parents (19, 22, 23, 27).

In all the included studies, the community engaged approach was appropriate because of the existing social networks among community partners that researchers used to spread information about cervical cancer and its prevention. Those community groups provided a platform for the research effort and provided a context for future implementation of community-based cervical cancer prevention strategies. While six studies did not utilize CBPR specifically, they employed some of the core principles of CBPR in an effort to identify and engage the target population through tribal leaders, religious leaders, and community health workers in order to increase knowledge about cervical cancer and increase screening utilization (19, 22, 23).

*Objective 2: Identify aspects of cervical cancer research where community engagement has been used*.

The articles included in this scoping review focused around three themes: assessing perceptions about cervical cancer, adapting existing cervical cancer screening strategies, and identifying and leveraging community partners.

In the first theme, researchers aimed to assess community perceptions about cervical cancer prevention. These articles sought to explore and understand the perceived need for, knowledge about, and motivations and preferences of receiving cervical cancer prevention services. The articles highlighted that there was minimal knowledge of cervical cancer among community members (18, 21, 27). These articles identified that community radio and local health talks were viable routes for information dissemination and attitude change (18). The authors found that the use of reputable community resources was a promising approach to increase awareness about and access to cervical cancer prevention services. Support from spouses and community leaders was also a key motivator in increasing utilization of cervical cancer prevention services (21). However, community stakeholders emphasized that health-seeking behavior occurs in a context shaped by economic, structural, and interpersonal conditions and health interventions need to address the multiple anxieties and lived experiences of the target group. These stakeholders stated that a narrow focus on the long-term risk for cervical cancer among adolescent girls has limited value (27).

The second theme focused on adapting current screening practices to meet community needs. In these studies, this included bringing cervical cancer screening out of the traditional clinic-based setting and into a community setting. This resulted in the development of Community Health Campaigns (CHCs), where researchers assessed acceptability, uptake and effectiveness of CHCs to increase cervical cancer screening (22, 26, 28). Studies found that CHCs improved cervical cancer screening rates but follow-up to treatment for those who tested positive was still a challenge (20, 22, 26).

The third theme found was that of identifying and leveraging community partners. The studies identified and utilized community resources such as traditional chiefs and church members to increase awareness and access to cervical cancer prevention resources (19, 23). Church members spearheaded workshops to increase cervical cancer awareness, and the influence of traditional Chiefs was leveraged to facilitate access to cervical cancer prevention services which increased access to cervical cancer screening and treatment (19, 23). Identifying and leveraging revered community leaders seems to elevate the importance of cervical cancer prevention in ways that are consistent with the community's shared values.

Most articles did not intentionally use the CBPR principles, but they were focused on engaging the community thus following some of the principles. The most used principles among all articles included: recognizing community as a unit of identity, and intentionally involving key stakeholders in the communities to address cervical cancer prevention. They also built on community strengths in increasing awareness and involving them in providing feedback on the development of cervical cancer prevention services.

*Objective 3: Describe the best practices for community engagement toward the end-goal of cervical cancer prevention and control*.

Based on reported outcomes from each of the included studies, the best practices for community engagement with the goal of cervical cancer prevention and control include: (1) working within existing community social support structures; (2) including members of the target community and their social networks in the implementation of the research effort; and (3) training those members to disseminate study findings to other members of those groups. Additionally, creating a social culture in which women are empowered to seek out and receive cervical cancer preventative services removed barriers that women face.

### The Use of CBPR Principles in Included Articles

*Principles 1 and 2: Recognize community as a unit of identity. Builds on strengths and resources within the community*.

The use of these two principals were explicitly outlined in eight of the nine included articles. These principles are the foundation for community engagement. The articles that described the community settings in which they conducted the study, the characteristics of the target population, and identified community partners (i.e., churches, local chiefs) that enabled them to engage the larger community were characterized as having described their use of these principles. See Table 2 for details.

**Table 2:** CBPR principles and principles used in included articles.

**CBPR Principle**	**Intent to use CBPR**	**Recognizes community as a unit of identity**	**Builds on strengths and resources within the community**	**Facilitates collaborative partnerships in all phases of the research**	**Integrates knowledge and action for mutual benefit of all partners**	**Promotes a co-learning and empowering process that attends to social inequities**	**Involves a cyclical and iterative process**	**Addresses health from both positive and ecological perspectives**	**Disseminates findings and knowledge gained to all partners**
Israel et al. Example		“Membership in a family, friendship network, or geographic neighborhood, are all socially constructed dimensions of identity, created and recreated through social interactions.”	“These may include skills and assets of individuals, networks of relationships characterized by trust, cooperation and mutual commitment, and mediating structures within the community such as churches and other organizations where community members come together—resources that have recently been referred to as social capital.”	“These partnerships focus on issues and concerns identified by community members, and create processes that enable all parties to participate and share influence in the research.”	“Community-based research may not always incorporate a direct-action component, but there is a commitment to the integration of research results with community change efforts with the intention that all involved partners will benefit.”	“Researchers learn from the knowledge and ‘local theories' of community members, and community members acquire further skills in how to conduct research.”	“A cyclical, iterative process that includes partnership development and maintenance, community assessment, problem definition, development of research methodology, data collection and analysis, interpretation of data, determination of action and policy implications, dissemination of results, action taking (as appropriate), specification of learnings, and establishment of mechanisms for sustainability.”	“Addresses the concept of health from a positive model that emphasizes physical, mental, and social well-being. It also emphasizes an ecological model of health that encompasses biomedical, social, economic, cultural, historical, and political factors as determinants of health and disease.”	“Seeks to disseminate findings and knowledge gained to all partners involved, in language that is understandable and respectful, and ‘where ownership of knowledge is acknowledged.”'
Abril et al. (18)									
Aja et al. (19)									
Awua et al. (20)									
Chigbu et al. (21)									
Huchko et al. (22)									
Kapambwe et al. (23)									
Mosavel et al. (24)									
Podolak et al. (25)									
Swanson et al. (26)									

*Principle 7: Addressing health from both positive and ecological perspectives*.

This principle was described in seven of the nine included articles. This principle highlights the importance of integrating the physical, mental, and social aspects of health with the biomedical, economic, and cultural aspects of health in the research process. The articles that explicitly described the use of this principle highlighted the importance of culture and social networks on cervical cancer outcomes and the role that those social networks can play in increasing knowledge about this disease in the community. See Table 2.

*Principles 4, 5, and 6: Integrates knowledge and action for mutual benefit of all partners. Promotes a co-learning and empowering process that attends to social inequities. Involves a cyclical and iterative process*.

These principles were described in six of the nine articles. These principles describe the practical aspects of an equitable relationship between the researchers and the communities where they work. Each of these six studies utilized some but not all principles in different ways, highlighting the differences in the types of relationships that researchers had established with their community members. See Table 2.

*Principle 3: Facilitates collaborative partnerships in all phases of the research*

This principle was described in three of the nine articles. The principle emphasizes collaboration between researchers and the community, a process that extends beyond simple participation in the research study toward active engagement in the development of the research question, the methods employed, and the community members who represent the community by participating in the study. See Table 2.

*Principle 8: Disseminates findings and knowledge gained to all partners*

This principle was not described in any of the included articles.

#### An Example of the Use of the CBPR Framework

Table 2 defines the eight principles of CBPR as described by Israel et al. in their seminal work (11). These principles effectively highlight areas in which traditional research fails to consider and/or include the community that they are aiming to serve. The principles are an important research guiding tool and a means to enhance community engagement practices in tangible ways, because they can aid in anticipating and addressing research limitations that are commonly observed when working in underserved populations before the research is completed. One article that was included in this scoping review gave a detailed account of their use of these CBPR principles. Examples of each principle has been taken from the study by Podolak et al. to illustrate how CBPR can be used in SSA communities and can be found in Supplementary Material.

#### Research Implications of Utilizing CBPR

As described above, CBPR is intended to be an iterative process which results in changes in the research question, design, and implementation. This iterative process ultimately leads to changes in the reported results as the process evolves. This phenomenon is evident in the articles that intentionally employed the CBPR framework. In the study by Mosvel et al., the use of CBPR resulted in the adjustment of their research question that prioritized community feedback. The research problem shifted from cervical cancer prevention to the broader concept of “Cervical Health,” which acknowledged that health seeking behavior is influenced by everyday stressors, including poverty, hunger, and violence (27). Through the CBPR process, the authors expressed that a focus on just cervical cancer was too narrow; highlighting the need for cervical cancer prevention programs to also cover other social determinants of health in order to generate demand from the community (27).

The study by Podolak et al. used CBPR to determine how local decision makers could apply participatory action research methods to make strategic decisions to effectively implement a cervical cancer prevention program. Researchers found that while community members expressed a desire for improved cervical cancer screening services, healthcare providers and suppliers of screening supplies had insufficient resources making it challenging to address the expressed community need. Community members also expressed the need for a subsidy to facilitate access to services, but it is unclear if these requests could be supported by the health system. The use of participatory action research in this study identified resource constraints on both the demand and supply of cervical cancer prevention services (25).

## Discussion

CBPR has been used in SSA communities to bolster cervical cancer prevention and control in the past. While not all studies included in this review intentionally used this framework, all included articles applied at least one of the traditional CBPR principles in their research process, which strengthened the receptivity of the research activity in the community. These studies illustrated that cervical cancer screening programs need to address factors related to limited access to services, such as limited resources that make it challenging for healthcare providers to provide screening services in a way that is acceptable to the community. These studies importantly highlight that community members often have other needs that often influence health seeking behavior, that should be addressed in conjunction with cervical cancer prevention efforts.

Community engagement and CBPR can be effective in achieving community-centered disease prevention and control research, more specifically, it can be used to address the lack of knowledge about cervical cancer in African communities. The studies included in this scoping review demonstrate different ways in which community engagement and CBPR specifically can be used throughout the research process. Overall, these articles demonstrate how utilizing community strengths and expertise can address previously identified issues, increase community uptake of education, increase screening utilization, and bridge the gap between healthcare providers, researchers, and the community at large. The findings of this review are consistent with previous CBPR studies that aimed to increase knowledge about cancer prevention and control in underserved communities living in the United States that experience disproportionate breast and prostate cancer burdens (13, 15).

### Challenges to Community Engagement in Research

There were several challenges and limitations with the implementation of the CBPR principles that arose across the studies. The first of these was language. Across both rural and urban settings, researchers encountered local dialects and languages that did not have a word for “cancer” (18, 27). This was also a challenge when local health workers were not proficient in the local language(s) of target study populations. This emphasizes the importance of community engagement, specifically when there are language and cultural barriers, and supports the need for partnerships between researchers, healthcare providers, and community members who can speak to those barriers as part of the research process. This approach not only improves the success of the research effort, but also empowers community members by enabling them to leverage their expertise in their culture and community to address their needs.

Another challenge is that the majority of rural women receive their information about women's health from other women in the community because this is their most accepted way of storytelling and information sharing (18). Frequently, the community can share misinformation on these topics guided by myths, misconceptions, and other personal beliefs. Therefore, women do not receive the most effective health information. However, this does identify an area in which an intervention can be applied to inform the community at large about a topic, and in this way have a more informed practice of storytelling-based information sharing. In fact, Abril et al. suggested utilizing radio as a medium to share cervical cancer education and other general health information because even the most rural of participants identified having access to radio and expressed that their communities gather in the evenings to listen to the radio news. Radio could be used to effectively communicate cervical cancer information to more rural communities, because this is already an accepted practice that is entrenched in their native language. Utilizing such widely available resources as radio news and constant education and awareness normalizes cervical cancer screening, with the goal of making seeking screening commonplace even in hard-to-reach communities.

Third, communication between health professionals and community members was hindered by limited access to technology. Studies were conducted in communities where participants had limited access to the internet, computers, or mobile phones (18). Additionally, some communities—particularly rural communities—experienced electricity outages further exacerbating communication challenges (18, 21). These conditions presented a challenge when determining the best format for participants to receive information about cervical cancer, screening availability, and their screening results. If immediate or same-day results were not available, the two methods used were text message notifications or home visits from community health workers. While some studies reported that the majority of women preferred to receive text notifications, they also cited that a large proportion of women did not own a cellphone, or they owned phone-call only cell phones that were primarily used for emergencies due to high cost (18, 26).

Related to communication challenges, another barrier cited in the studies was loss to follow-up. Factors that contributed to loss to follow-up were the location of screening services, whether multiple visits were required between screening, results, and treatment, and the results delivery method chosen by participants. One article suggested health fairs that were planned around seasonal activities, thereby providing an occasion for healthcare providers to interact with community members outside of the traditional healthcare setting (25). This is an area for future research as improving methods of communication between healthcare providers and their patients could play a pivotal role in down staging cervical cancer diagnoses in underserved populations, especially among those living in rural and hard-to-reach locations.

Across all the studies, engaging local community leaders—chiefs, pastors, educators, and other leaders—yielded favorable results for increasing knowledge and use of cancer screening services. Interestingly, studies found that husbands also played an influential role in determining whether women would get screened. Married women would often need their husband's permission and this was sometimes the reason women declined to participate in the cancer screening activities (18). They further explained that the vast majority of husbands were in agreement with screening, therefore women might also use this as a way to indirectly express their screening hesitancy (18). Many of the articles included in this review focused almost exclusively on increasing knowledge about cervical cancer and increasing utilization of screening services among women. However, this review has highlighted the importance that men, specifically husbands, have on women's decision-making process in this arena. Consequently, further research on knowledge about and acceptability of cervical cancer screening among men is necessary to provide further context about the sociocultural influences on cervical cancer screening utilization among women.

For most studies that were focused on feasibility, the engagement of community members was beneficial. While this approach was effective, this also emphasizes the need for studies that are focused on implementing evidence-based strategies for decreasing disparities related to incidence and mortality from cervical cancer in sub-Saharan Africa. As this review has highlighted, community engagement through the empowerment of existing social networks is a promising strategy for achieving community-wide impact. While it is unclear how lasting that impact might be, this is a step toward addressing persistent cancer disparities. These findings can benefit other LMICs that struggle with similar challenges to addressing disparities due to the barriers described above.

### Review Limitations

Though this review had several strengths, it also had notable limitations. The first is that a broad definition of community engagement was used to capture all types of research that was being conducted. This broad definition falls outside of the scope of traditional CBPR but was beneficial to this review as a holistic picture of community engagement in sub-Saharan Africa was captured. Another limitation in this review process was that the included articles varied widely in all aspects of the research that it was difficult to synthesize the results. Due to the highly individualized nature of community-engaged work with relation to health, and the additional cost of publication, it is unlikely that efforts of grass-roots organizations that are conducting interventions in their communities relating to cervical cancer prevention and control were captured.

This review also presents an important question about how researchers quantify the level of community engagement they have achieved in their study. This review utilized the principles described by Israel et al., in their seminal work, as it has become the foundation of this field of research, however this approach was flawed because it relied on the subjective evaluation of the review team, and the information that was described in the included research articles. As the field of community engaged research develops, it will become increasingly important to have an objective measure of engagement even though this process can be tailored to a specific community.

### Conclusions

This review is the first step toward filling the gap in the literature about contexts in which community engagement can be utilized to address cancer related disparities. The various methods of community engagement that have been used by previous researchers, the challenges that they faced, and future directions for the use of community engagement in SSA communities were captured. To our knowledge, there is one other review on cervical cancer prevention in sub-Saharan Africa, but it does not focus exclusively on community engagement in order to address disparities pertaining to cervical cancer incidence and mortality (29). It was demonstrated that CBPR and community engaged research practices in general can be used in SSA communities to increase awareness about and utilization of cervical cancer screening services.

Our results highlight that because cultural context is central to uptake of services of behavior change, identifying and partnering with community networks is essential for the success of any research effort that aims to change perceptions and behavior of the community. This review highlights that community engagement as a tool for health disparities research has been underutilized, and that results in the persistence of disproportionate cervical cancer mortality suffered by women in sub-Saharan Africa. The intentional identification and development of equitable and collaborative partnerships between institutions, healthcare providers, and community members to address cancer related health disparities is the next necessary step toward addressing the needs of underserved community members living in sub-Saharan Africa and other communities globally.

## Author Contributions

MH, JM, and AS formulated and defined the research question. MH, LK, NM, and DV reviewed abstracts, titles, and full texts included in the search. DJM, MH, and PM developed the search strategies for the databases included in the search. MH, JM, EJ, and PM addressed reviewer comments. All authors contributed to writing the manuscript.

## Conflict of Interest

The authors declare that the research was conducted in the absence of any commercial or financial relationships that could be construed as a potential conflict of interest.

## Abbreviations

LMIC: Low-and-middle-income countries
SSA: Sub-Saharan Africa
CBPR: Community-based participatory research
HPV: human papillomavirus
CHCs: Community Health Campaigns.
