# Supplementary material for: Community-Engaged Approaches to Cervical Cancer Prevention and Control in Sub-Saharan Africa: A Scoping Review
Source: Front Glob Womens Health. 2021 Jul 19;2:697607. doi: 10.3389/fgwh.2021.697607 (PMC8594022; doi:10.3389/fgwh.2021.697607)
Supplement: Supplementary file 2 [file Data_Sheet_2.docx]

Supplementary Material

# PubMed Search String

The PubMed search we conducted is below:

("participatory research" OR "participatory action research" OR "community participation" OR "community engagement" OR "community engaged" OR "community health partnership" OR "community health partnerships" OR "community academic partnership" OR "community academic partnerships" OR "community partnership" OR "community partnerships" OR "research partnership" OR "research partnerships" OR "community-based participatory research"[Mesh] OR "community participation"[Mesh] OR "community-institutional relations"[Mesh] OR "cooperative behavior"[Mesh])

**AND**

("Africa south of the Sahara" OR "Central Africa" OR Cameroon OR "Central African Republic" OR Chad OR Congo OR "Democratic Republic of the Congo" OR "Equatorial Guinea" OR Gabon OR "Sao Tome and Principe" OR "Eastern Africa" OR Burundi OR Djibouti OR Eritrea OR Ethiopia OR Kenya OR Rwanda OR Somalia OR "South Sudan" OR Sudan OR Tanzania OR Uganda OR "Southern Africa" OR Angola OR Botswana OR Lesotho OR Malawi OR Mozambique OR Namibia OR "South Africa" OR Swaziland OR Zimbabwe OR "Western Africa" OR Benin OR "Cabo Verde" OR "Cape Verde" OR "Cote d'Ivoire" OR Gambia OR Ghana OR Guinea OR "Guinea-Bissau" OR Liberia OR Mali OR Mauritania OR Niger OR Nigeria OR Senegal OR "Sierra Leone" OR Togo OR "Africa south of the Sahara"[MeSH] OR "Africa, central"[MeSH] OR "Central African Republic"[MeSH] OR "Democratic Republic of the Congo"[MeSH] OR "Equatorial Guinea"[MeSH] OR "Sao Tome and Principe"[MeSH] OR "Africa, Eastern"[MeSH] OR "South Sudan"[MeSH] OR "Africa, Southern"[MeSH] OR "South Africa"[MeSH] OR "Africa, Western"[MeSH] OR "Cabo Verde"[MeSH] OR "Cote d'Ivoire"[MeSH] OR "Guinea-Bissau"[MeSH] OR "Sierra Leone"[MeSH])

**AND**

(Uterine cervical cancer OR uterine cervical neoplasms OR cancer of the cervix OR cervical cancer OR "uterine cervical neoplasms"[mesh])
