# Supplementary material for: Community-Engaged Approaches to Cervical Cancer Prevention and Control in Sub-Saharan Africa: A Scoping Review
Source: Front Glob Womens Health. 2021 Jul 19;2:697607. doi: 10.3389/fgwh.2021.697607 (PMC8594022; doi:10.3389/fgwh.2021.697607)
Supplement: Supplementary file 3 [file Data_Sheet_3.docx]

Supplementary Material

# An Example of the Use of the CBPR Framework

*Principle 1: Recognize community as a unit of identity.*

“The PI developed a discussion guide in collaboration with the Project Management Team to ensure that sensitivity to local values and culture were addressed when conducting semi-structured interviews and focus groups with urban and rural women[...]Participants were asked to provide their perspectives in response to two statements: tell me about your experiences with CC, and tell me about those things (good or bad) that you think would have an impact on the implementation of a cervical self-sampling program. During the interviews and focus groups, the PI (through the translator) articulated her interpretation of what participants described to better validate the participants' meaning. This process of concurrent member validation aided in ensuring that the PI appropriately understood the participants comments." (Podolak p3)

*Principle 2: Builds on strengths and resources within the community.*

 “By including local experts (with the required tacit, explicit, and cultural knowledge) in the decision-making process, ownership of decisions could be strengthened, thereby increasing the probability that they will be acted upon." (Podolak p2)

*Principle 3: Facilitates collaborative partnerships in all phases of the research.*

“To ensure data quality regarding the composite descriptions, the PI reviewed the outputs from the phenomenological assessment with Project Management Team and Local Decision Influencing Participant members in a workshop setting and requested their feedback...Upon review of these outputs and engaging in open dialogue, the Project Team collectively decided on how this information pertaining to social acceptability would be addressed in the design of the CSSP. This power sharing relationship, in which decisions were made collectively, contributed to a more comprehensive CSSP design." (Podolak p4)

*Principle 4: Integrates knowledge and action for mutual benefit of all partners.*

 “The Principle Investigator, Project Management Team and Local Decision Influencing Participant members decided to be consistent with the [Participatory Action Research] methodology and operate as co-decision makers.” (Podolak p3)

*Principle 5: Promotes a co-learning and empowering process that attends to social inequalities.*

“Phenomenology description involved determining the essence of women's perceptions of CC and self-sampling, i.e., identifying those factors that would predispose a woman to adopt or reject collecting her own cervical sample.” (Podolak p3)

*Principle 6: Involves a cyclical and iterative process.*

“Consistent with the qualitative research paradigm of constructivism and [Participatory Action Research], data collection and analysis was a collaborative, participative, reflective and iterative process. Repeated rounds of data collection and analysis took place before, during and after four Workshop events.” (Podolak p4)

*Principle 7: Addresses health from both positive and ecological perspectives.*

“Scenario building involved creating potential scenarios that described different future states for the CSSP in Kenya. Additional data for each potential scenario was collected to produce plausible descriptions of how a CSSP could be implemented, followed by an impact analysis to determine the consequences of each scenario.”  (Podolak p3)

*Principle 8: Disseminates findings and knowledge gained to all partners.* Not described.

Only two other articles intentionally used the CBPR framework as the foundation for their research efforts^20,24^. Though the researchers in the Podolak et al. article provided examples of nearly all CBPR principles, they did not describe the process they might have used to disseminate findings between all partners. This is an example of one way that traditional research fails to impact the community simply because the community is not informed of the findings of the project. This presents a challenge for sustainability of research interventions.
